# Involvement of the OB-fold and the C-terminal acidic tip of SSB in its interaction with RecO from *Thermus thermophilus* HB8

**DOI:** 10.1093/jb/mvag042

**Published:** 2026-06-16

**Authors:** Kazuki Kobayashi, Masao Inoue, Kenji Fukui, Riku Aono, Anna Ochi, Ryoji Masui, Hisaaki Mihara

**Affiliations:** College of Life Sciences, Ritsumeikan University, 1-1-1 Nojihigashi, Kusatsu, Shiga 525-8577, Japan; College of Life Sciences, Ritsumeikan University, 1-1-1 Nojihigashi, Kusatsu, Shiga 525-8577, Japan; R-GIRO, Ritsumeikan University, 1-1-1 Nojihigashi, Kusatsu, Shiga 525-8577, Japan; Department of Food Science and Nutrition, Nara Women’s University, Kita-Uoya-Nishimachi, Nara, Nara 630-8506, Japan; College of Life Sciences, Ritsumeikan University, 1-1-1 Nojihigashi, Kusatsu, Shiga 525-8577, Japan; College of Life Sciences, Ritsumeikan University, 1-1-1 Nojihigashi, Kusatsu, Shiga 525-8577, Japan; Graduate School of Science, Osaka Metropolitan University, 3-3-138 Sugimoto, Sumiyoshi-ku, Osaka 558-8585, Japan; College of Life Sciences, Ritsumeikan University, 1-1-1 Nojihigashi, Kusatsu, Shiga 525-8577, Japan

**Keywords:** homologous recombination, protein–protein interaction, recombination mediator, single-stranded DNA-binding protein*; Thermus thermophilus* HB8

## Abstract

In bacterial homologous recombination, the single-stranded DNA (ssDNA)–binding protein (SSB) coats exposed ssDNA to protect it but simultaneously inhibits RecA filament nucleation. RecO, a recombination mediator, overcomes this inhibition by interacting with SSB and displacing it from ssDNA. This SSB–RecO interaction has long been considered to rely primarily on the conserved C-terminal acidic tip (C-tip) of SSB. Here, using domain-truncated SSB variants from *Thermus thermophilus* HB8, we show that RecO directly binds both the SSB oligonucleotide/oligosaccharide-binding fold (OB-fold) and the C-tip. These two contacts make distinct contributions to RecO’s mediator functions. Arg127 in RecO contributes to recognition of the SSB C-tip and to double-stranded DNA (dsDNA) binding, the latter of which is suppressed by the SSB C-tip. In RecO-mediated ssDNA annealing, the SSB C-tip primarily modulates SSB dynamics on ssDNA rather than directly activating RecO. Moreover, stable assembly of the SSB–RecO–RecR ternary complex requires the SSB C-tip. Our findings support a mechanism in which the SSB C-tip modulates RecO function and governs SSB behaviour on ssDNA, whereas the OB-fold provides additional contacts whose functional contributions remain to be elucidated.

## Graphical Abstract

**Graphical Abstract:**
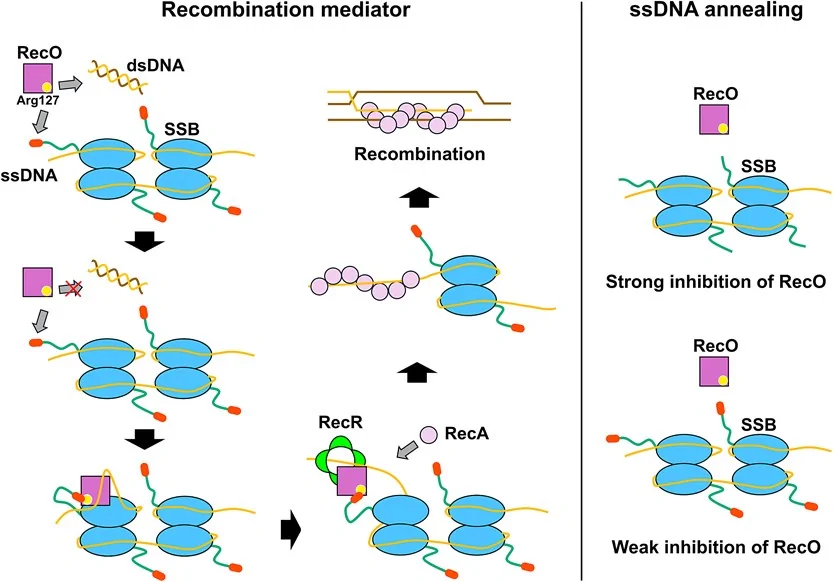

## Abbreviations

C-tip: C-terminal acidic tip
dsDNA: double-stranded DNA
IDL: intrinsically disordered linker
nt: nucleotide
OB-fold: oligonucleotide/oligosaccharide-binding fold
pI: isoelectric point
SSB: single-stranded DNA-binding protein
ssDNA: single-stranded DNA
WT: wild type

Homologous recombination is a central pathway for the accurate repair of double-strand breaks and stalled replication forks and is conserved across all three domains of life *(**1–4**)*. In bacteria, the RecA recombinase orchestrates the homology search and strand exchange during homologous recombination *(**5**)*. Formation of RecA nucleoprotein filaments on single-stranded DNA (ssDNA) depends on mediator proteins that displace the ssDNA-binding protein (SSB), which coats ssDNA *(**6**,*  *7**)*. SSB protects ssDNA from nucleases and suppresses the formation of unfavourable secondary structures yet simultaneously impedes RecA nucleation. An SSB monomer consists of one or two oligonucleotide/oligosaccharide-binding fold (OB-fold) domains that bind ssDNA, a C-terminal acidic tip (C-tip) that interacts with various proteins and an intrinsically disordered linker (IDL) that connects these two regions *(**8**)*. SSB is highly conserved across bacteria, but its oligomeric state varies among species: in *Escherichia coli*, SSB assembles into a homotetramer *(**9**)*, whereas in species such as *Deinococcus radiodurans* and *Thermus thermophilus*, it forms a homodimer *(**10**)*, suggesting diversification among organisms.

There are two principal presynaptic pathways that deliver RecA to ssDNA: the RecBCD pathway, which processes double-strand break ends to produce 3′-ssDNA tails and directly loads RecA *(**11**)*, and the RecOR/RecFOR pathway, which targets both RecJ-processed 3′-ssDNA ends and gapped ssDNA–double-stranded DNA (dsDNA) junctions to nucleate RecA filaments *(**6**,*  *7**,*  *12**,*  *13**)*. In the RecOR/RecFOR pathway, RecO forms a complex with RecR to facilitate RecA loading onto ssDNA and further associates with RecF to assemble a ternary complex that promotes RecA loading at ssDNA–dsDNA junctions *(**7**,*  *13**)*. RecO possesses both ssDNA- and dsDNA-binding activities and acts as a central mediator by displacing SSB from ssDNA, thereby enabling ssDNA annealing and RecA-catalysed homologous recombination. Displacement of SSB from ssDNA by RecO is mediated by direct interactions with both SSB and ssDNA; however, the mode of interaction between RecO and SSB varies across species *(**7**,*  *14**)*. While dsDNA-binding activity of *E. coli* and *D. radiodurans* RecO has been demonstrated *(**7**,*  *15**,*  *16**)*, how this activity relates to the role of RecO as a recombination mediator remains unclear. Moreover, to our knowledge, the dsDNA-binding activity of *T. thermophilus* RecO has not been reported.

*Escherichia coli* SSB recruits RecO to ssDNA via a direct interaction between its C-tip and a hydrophobic pocket on RecO *(**7**)*. By contrast, *D. radiodurans* RecO, which shares low amino acid sequence identity (~20%) with *E. coli* RecO, shows only weak interaction with the SSB C-tip peptide in the presence of RecR. In *T. thermophilus* RecO, which also shows ~20% amino acid sequence identity with *E. coli* RecO, an NMR study has shown that the SSB C-tip directly binds to Arg127 of RecO *(**14**)*. This residue is indispensable for formation of the SSB–RecO complex and for the ssDNA annealing activity of RecO. However, distinct patterns of chemical-shift perturbation have been observed in RecO upon titration with full-length SSB, compared with the synthetic C-tip peptide, suggesting interaction sites beyond the C-tip.

In the present study, we analysed *T. thermophilus* SSB–RecO interactions in detail and characterized the direct interaction between the SSB OB-fold and RecO. Moreover, we demonstrated that the SSB C-tip contributes to RecO’s ssDNA annealing and dsDNA-binding activities, as well as to the formation of the SSB–RecO–RecR ternary complex. Our results provide mechanistic insight into how RecO displaces SSB via coordinated interactions with both the SSB OB-fold and C-tip.

## Materials and Methods

### Construction of expression plasmids

The expression plasmids for *T. thermophilus* SSB (ttSSB) (pET-11a/tt*ssb*), ttRecO (pET-11a/tt*recO*) and ttRecR (pET-11a/tt*recR*) were obtained from the RIKEN BioResource Research Center (Tsukuba, Japan), which were constructed by ligating the amplified *ttha0244*, *ttha0623* and *ttha1600* fragments (GenBank, GCA_000091545.1), respectively, into the NdeI/BamHI sites of the pET-11a vector. The expression plasmid for His_6_-tagged ttSSB (pET-HisTEV/tt*ssb*) was constructed by inserting the amplified gene fragment from pET-11a/tt*ssb* into the NdeI/EcoRI sites of the pET-HisTEV vector *(**17**)*. To construct the pET-HisTEV-based expression plasmid for the domain-truncated mutant, ttSSBΔC17 (amino acid residues 1–246), pET-11a/tt*ssbΔC17* was constructed via the inverse PCR procedure using pET-11a/tt*ssb* as a template, and then, the amplified DNA fragment encoding ttSSBΔC17 was inserted into the NdeI/EcoRI sites of the pET-HisTEV vector. The expression plasmids for ttSSBΔIDL (amino acid residues 1–228 and 247–263) and ttSSBΔIDLΔC17 (amino acid residues 1–228) were constructed by the inverse PCR procedure using pET-HisTEV/tt*ssb* as a template. The expression plasmid for the ProS2-fused ttSSB (pCold-ProS2TEV/tt*ssb*) was constructed by inserting the amplified gene fragment from pET-HisTEV/tt*ssb* into the NdeI/XbaI site of the pCold-ProS2TEV vector, which was constructed based on pCold-ProS2 (Takara Bio, Shiga, Japan) by introducing a DNA fragment encoding a TEV protease cleavage site (ENLYFQS) immediately downstream of the Factor Xa cleavage site. The expression plasmids for the ProS2-OB_ttSSB_ (amino acid residues 1–228) and ProS2-C17_ttSSB_ (amino acid residues 247–263) were constructed by inverse PCR using pCold-ProS2TEV/tt*ssb* as a template. The expression plasmid for ProS2-IDL_ttSSB_ (amino acid residues 229–246) was constructed by inserting the amplified DNA fragment into the NdeI/XbaI site of the pCold-ProS2TEV vector. To construct the expression plasmid for the ttRecO^R127A^ mutant, the site-directed mutagenesis was performed via the PrimeSTAR mutagenesis procedure (Takara Bio) using pET-11a/tt*recO* as a template. All primer sequences used for plasmid construction are listed in Table S1.

### Protein overexpression and purification

*E. coli* C43(DE3) cells (Lucigen, Middleton, WI, USA) were transformed with pET-HisTEV/tt*ssb*. Transformants were cultured for 20 h at 37°C in 1 L of an overexpression medium *(**17**)* containing 156 mM sodium/potassium phosphate (pH 7.3), 1.6% tryptone (Nacalai Tesque, Kyoto, Japan), 1.0% yeast extract (Nacalai Tesque), 0.5% NaCl, 0.75% glycerol, 0.05% glucose, 0.2% lactose monohydrate and 100 μg/ml ampicillin. The cells were harvested by centrifugation, resuspended in buffer I (50 mM Tris–HCl (pH 7.5) and 500 mM NaCl) and lysed by ultrasonication on ice. The lysate was heat-treated at 65–70°C for 5 min. After centrifugation (20,000 × *g*) for 10 min at 4°C, the supernatant was loaded onto a TALON Superflow Metal Affinity Resin (Takara Bio) pre-equilibrated with buffer I. ttSSB protein was eluted by a stepwise increase in the imidazole concentration (30–500 mM) in buffer I. Fractions containing ttSSB were pooled, and the protein was precipitated with 1.6 M ammonium sulfate. After centrifugation (15,000 × *g*) for 10 min at 4°C, the pellet was dissolved in 50 mM Tris-HCl (pH 7.5) containing 2 mM EDTA and then, the solution was loaded onto a TOYOPEARL AF-Heparin HC-650 M (TOSOH, Tokyo, Japan) pre-equilibrated with 50 mM Tris-HCl (pH 7.5). ttSSB was eluted by a stepwise increase in NaCl concentration (50–500 mM) in 50 mM Tris-HCl (pH 7.5). The eluted protein was concentrated and buffer-exchanged into buffer I using an Amicon Ultra (Merck, Darmstadt, Germany). The ttSSB mutants (ttSSBΔIDL, ttSSBΔC17 and ttSSBΔIDLΔC17) were overexpressed and purified using the same procedures as for ttSSB.

*E. coli* DH5α cells (TOYOBO, Osaka, Japan) were transformed with pCold-ProS2TEV*/*tt*ssb*. Transformants were cultured at 37°C in 1 L of lysogeny broth medium supplemented with 100 μg/ml ampicillin. When the optical density at 600 nm reached ~0.5, the culture was cooled on ice for 30 min. Protein expression was induced by adding 0.1 mM isopropyl β-d-thiogalactopyranoside (Protein Ark, Sheffield, UK), followed by incubation at 16°C for 20 h. Cell harvest, lysis, heat treatment, chromatography on TALON Superflow Metal Affinity Resin and ammonium sulfate precipitation were performed using the same procedure as for ttSSB. After ammonium sulfate precipitation, the pellet was dissolved in buffer I, and then, the solution was loaded onto a Superdex 200 Increase 10/300 GL column (Cytiva, Marlborough, MA, USA) pre-equilibrated with buffer I. The eluted ProS2-ttSSB was concentrated and buffer-exchanged into buffer II (50 mM Tris-HCl (pH 7.5) and 100 mM NaCl) using an Amicon Ultra. The other ProS2-fused proteins (ProS2, ProS2-OB_ttSSB_, ProS2-IDL_ttSSB_ and ProS2-C17_ttSSB_) were overexpressed and purified following the same procedures.

*E. coli* Rosetta2(DE3) cells (Novagen, Madison, WI, USA) were transformed with pET-11a/tt*recO*. Transformants were cultured for 20 h at 37°C in 1 L of the overexpression medium as described above. Cell harvest, lysis and heat treatment were performed using the same procedure as for ttSSB. After centrifugation, the supernatant was diluted to 100 mM NaCl and loaded onto a SP Sepharose Fast Flow (Cytiva) pre-equilibrated with buffer II. ttRecO was eluted by a stepwise increase in NaCl concentration (0.2–1 M) in 50 mM Tris-HCl (pH 7.5). The eluted ttRecO was then loaded onto the Superdex 200 Increase 10/300 GL column equilibrated with 50 mM Tris-HCl (pH 7.5) and 2 M NaCl. The eluted ttRecO was concentrated and buffer-exchanged into buffer I using an Amicon Ultra (Merck). ttRecO^R127A^ was overexpressed and purified following the same procedures.

*E. coli* Rosetta2(DE3) cells were transformed with pET-11a/tt*recR*. The cells were cultured for 20 h at 37°C in 1 L of the overexpression medium as described above. The cells were harvested by centrifugation, resuspended in the buffer (50 mM Tris–HCl (pH 7.5) and 1 mM phenylmethanesulfonyl fluoride) and lysed by ultrasonication on ice. The lysate was heat-treated at 70–75°C for 10 min. After centrifugation (39,000 × *g*) for 60 min at 4°C, ammonium sulfate was added to the supernatant to a final concentration of 1.3 M. The solution was loaded onto a TOYOPEARL Phenyl-650 M (TOSOH) pre-equilibrated with buffer containing 50 mM Tris-HCl (pH 7.5) and 1.3 M ammonium sulfate. The column was washed with the buffer and eluted with a linear gradient of 1.3–0 M ammonium sulfate in 50 mM Tris-HCl (pH 7.5). Fractions containing ttRecR were pooled, diluted 5-fold with 50 mM Tris-HCl (pH 7.5), and loaded onto a TOYOPEARL SuperQ-650 M (TOSOH) pre-equilibrated with 50 mM Tris-HCl (pH 7.5). The column was washed with 50 mM Tris-HCl (pH 7.5) and eluted with a linear gradient of 0–0.6 M NaCl in 50 mM Tris-HCl (pH 7.5). Fractions containing ttRecR were pooled and the protein was precipitated with 2.0 M ammonium sulfate. After centrifugation (39,000 × *g*) for 60 min at 4°C, the pellet was dissolved in buffer containing 50 mM Tris-HCl (pH 7.5), 100 mM NaCl and 0.1 mM ZnSO_4_, and then, the solution was loaded onto a HiLoad 16/60 Superdex 200 pg column (Cytiva) in buffer II. The eluted ttRecR was concentrated and buffer-exchanged into buffer containing 50 mM Tris-HCl (pH 7.5) and 150 mM NaCl using a Vivaspin concentrator (Sartorius AG, Göttingen, Germany).

The protein concentration was determined on the basis of the absorbance at 280 nm using the molar extinction coefficients (M^−1^ cm^−1^) calculated by the previously described procedure *(**18**)*. In the proteins without tryptophan, the molar extinction coefficient was estimated from the absorbance at 274 nm and considering each tyrosine residue contributes 1400 M^−1^ cm^−1^. The values are as follows: 42,000 (at 280 nm) for ttSSB, ttSSBΔIDL, ttSSBΔC17 and ttSSBΔIDLΔC17; 9800 (at 274 nm) for ProS2, ProS2-IDL_ttSSB_ and ProS2-C17_ttSSB_; 50,820 (at 280 nm) for ProS2-ttSSB and ProS2-OB_ttSSB_; 20,370 (at 280 nm) for ttRecO and ttRecO^R127A^; and 8400 (at 274 nm) for ttRecR. The purity of each protein was assessed by SDS-PAGE (Fig. S1). Small aliquots of the final protein preparations were flash-frozen in liquid nitrogen and stored at −80°C.

### Native agarose gel electrophoresis analysis

Protein mixtures containing ttSSB or its derivatives (5 μM) and ttRecO or ttRecO^R127A^ (0–15 μM) were incubated at 37°C for 15 min in buffer containing 90 mM Tris-HCl (pH 7.5), 100 mM NaCl, 10% glycerol and 0.01% bromophenol blue. Reaction mixtures (20 μl each) were loaded onto a 0.5% PrimeGel Agarose GOLD 3-40 K (Takara Bio) and then electrophoresed in 1× TAE buffer (40 mM Tris-acetate and 1 mM EDTA, pH 8.3). Gels were stained with Coomassie Brilliant Blue G-250. As a control, hen egg-white lysozyme (15 μM; Wako, Osaka, Japan) was used in place of ttRecO.

### DNA electrophoretic mobility shift assay

For dsDNA-binding assays of ttRecO, a 100-bp dsDNA fragment was amplified by PCR from the ampicillin-resistance gene of pUC119. The primer sequences used for dsDNA construction are listed in Table S1. dsDNA (2 μM in bp), ttRecO (0–4 μM) or ttRecO^R127A^ (0–8 μM) and either ttSSB or its derivatives (0–8 μM) or a synthetic ttSSB C-tip peptide (0–32 μM; DIDEGLEDFPPEEELPF, 97% purity; GenScript, Piscataway, NJ, USA) were incubated at 37°C for 15 min in buffer containing 50 mM Tris-HCl (pH 7.5), 50 mM NaCl, 0.05 mg/ml bovine serum albumin, 10% glycerol and 0.01% bromophenol blue. Reaction mixtures were prepared by first adding the buffer, followed by independent addition of protein/peptide and DNA solutions without premixing. Reactions were initiated by brief centrifugation to ensure simultaneous mixing of all components. For ssDNA-binding assays of ttSSB, a 60-mer oligo(dT) (300 nM) and ttSSB or its derivatives (0–8 μM) were incubated at 37°C for 15 min in a buffer containing 50 mM Tris-HCl (pH 7.5), 50 mM NaCl, 0.05 mg/ml bovine serum albumin, 10% glycerol and 0.01% bromophenol blue.

Reaction mixtures (10 μl each) were loaded onto a 4–15% gradient polyacrylamide gel and then electrophoresed in 0.5× TBE buffer (49 mM Tris-borate and 2 mM EDTA, pH 8.4). Gels were stained with GelRed Nucleic Acid Gel Stain (Biotium, Fremont, CA, USA), and bands were visualized by an Odyssey XF Imaging System (LI-COR Biotechnology, Lincoln, NE, USA). Band intensities were quantified with Image Studio software (version 2.0; LI-COR Biotechnology). The percentage of bound dsDNA was calculated as: Bound dsDNA (%) = 100 × (1 − *S*_free_/*S*_control_), where *S*_control_ is the dsDNA signal intensity in protein-free control and *S*_free_ is the free dsDNA signal intensity in each lane. The percentage of free dsDNA (%) was calculated as: Free dsDNA (%) = 100 × *S*_free_/*S*_control_.

### Native PAGE analysis

Protein mixtures containing ProS2-ttSSB or its derivatives (5 μM), ttRecO (15 μM) and ttRecR (0–30 μM) were incubated at 37°C for 15 min in buffer containing 90 mM Tris-HCl (pH 7.5), 100 mM NaCl, 10% glycerol and 0.01% bromophenol blue. Reaction mixtures (20 μl each) were analysed by native PAGE on 8% polyacrylamide gels run in Tris-glycine buffer (25 mM Tris and 192 mM glycine, pH 8.3). The gel was stained with Coomassie Brilliant Blue G-250. Bands of interest were excised, finely minced, treated with SDS sample buffer, and heated at 95°C for 5 min. Samples were then analysed by SDS-PAGE on a 15% gel and stained using a SilverQuest Silver Staining Kit (Invitrogen, Carlsbad, CA, USA).

### s‌sDNA annealing assay

The ssDNA substrate was prepared by digesting pUC119 with EcoRI, heat-denaturing at 100°C for 5 min and rapid cooling in ice water. The heat-denatured linearized pUC119 dsDNA (15 μM in nucleotides, nt) and ttSSB or its derivatives (1 μM) were incubated in 10 μl of buffer containing 45 mM Tris-HCl (pH 7.5) and 45 mM NaCl at 37°C for 5 min. The ssDNA annealing reaction was initiated by adding 1 μl of 10 μM ttRecO or ttRecO^R127A^. To stop the reaction at the indicated times, 1 μl of 5% SDS and 1 μl of 20 mg/ml proteinase K (Takara Bio) were added to the reaction mixtures, which were then incubated at 4°C for 30 min. Samples were electrophoresed on a 1% Agarose S (NIPPON GENE, Tokyo, Japan) in 1× TAE buffer. Gels were stained with a GelRed Nucleic Acid Gel Stain, and DNA bands were visualized using an Odyssey XF Imaging System. Band intensities were quantified with Image Studio software. The percentage of annealed product was calculated as: Product (%) = 100 × *S*_dsDNA_/(*S*_ssDNA_ + *S*_dsDNA_), where *S*_ssDNA_ and *S*_dsDNA_ are the ssDNA and dsDNA signal intensities in each lane, respectively.

To reproduce the conditions reported previously *(**14**)*, the heat-denatured linearized pUC119 dsDNA (15 μM in nt) and ttSSB (1 μM) were incubated in 10 μl of buffer containing 45 mM Tris-HCl (pH 7.5), 5 mM NaCl, 10 mM MgCl_2_, 1 mM DTT and 50 mM KCl at 37°C for 5 min. The ssDNA annealing reaction was initiated by adding 1 μl of 10 μM ttRecO or ttRecO^R127A^. To stop the ssDNA annealing reaction, 1 μl of 5% SDS and 1 μl of 20 mg/ml proteinase K were added, followed by incubation at 4°C for 5 min and then at 37°C for 15 min to complete deproteinization. Samples were electrophoresed on a 1% agarose gel in 1× TAE buffer.

## Results

### Both the OB-fold and the C-tip of ttSSB contribute to ttRecO binding

**Fig. 1:**
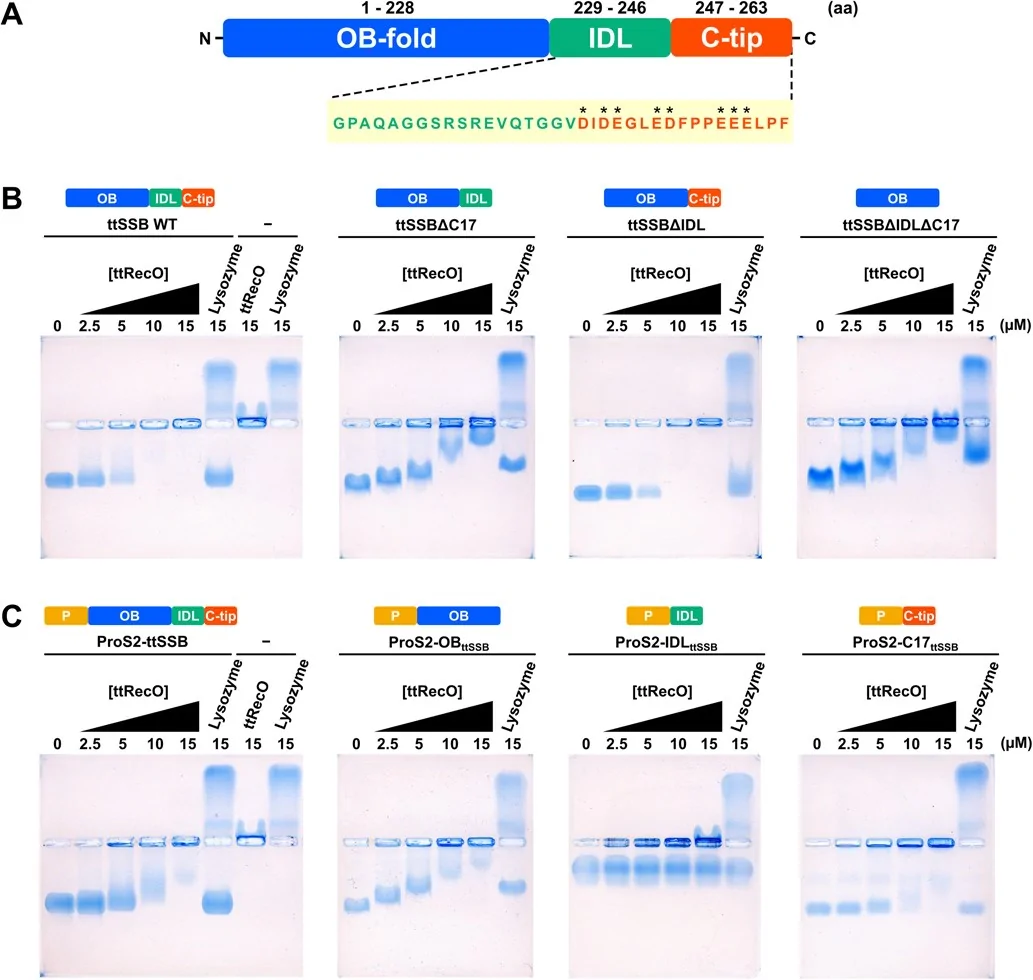
**Interactions between *Thermus thermophilus* single-stranded DNA binding protein (ttSSB) derivatives and ttRecO**. (A) Schematic of ttSSB domain organization: the oligonucleotide/oligosaccharide-binding fold (OB-fold), blue; the intrinsically disordered linker (IDL), green; and the C-terminal acidic tip (C-tip), orange. The amino acid sequences of the IDL and the C-tip are shown. Asterisks indicate the negatively charged residues in the C-tip. (B, C) Native agarose gel electrophoresis of the ttSSB–ttRecO interactions. ttSSB wild type (WT) and the domain-truncated mutants (ttSSBΔC17, ttSSBΔIDL and ttSSBΔIDLΔC17) were analysed in (B), whereas ProS2-fused proteins (ProS2-ttSSB, ProS2-OB_ttSSB_, ProS2-IDL_ttSSB_, ProS2-C17_ttSSB_) were analysed in (C). The concentration of ttSSB WT and its derivatives was 5.0 μM, and ttRecO was added at the indicated concentrations. Lysozyme (15 μM) was used as a negative control. Schematic representations of the domain organization of each ttSSB derivative are shown above each gel as indicated in (A). ProS2-tag (P) is shown in yellow.

To elucidate the structural regions of ttSSB responsible for interaction with ttRecO, we constructed His_6_-tagged, the domain-truncated mutants: ttSSBΔC17 lacking the C-tip (amino acid residues 247–263), ttSSBΔIDL lacking the IDL region (amino acid residues 229–246) and ttSSBΔIDLΔC17 lacking both regions (Fig. 1A and Fig. S1).

Interactions between ttSSB derivatives and ttRecO were evaluated by native agarose gel electrophoresis. In this assay, samples are loaded into wells at the centre of the gel and separated according to their net charge (Fig. 1B). In 1× TAE buffer (pH 8.3), the acidic proteins—ttSSB (isoelectric point (pI) = 5.67), ttSSBΔC17 (pI = 7.17), ttSSBΔIDL (pI = 5.56) and ttSSBΔIDLΔC17 (pI = 6.77)—migrated towards the anode, whereas the basic proteins ttRecO (pI = 10.3) and hen egg-white lysozyme (pI = 9.36; negative control) migrated towards the cathode. Protein–protein interactions were detected by mobility shifts of the corresponding bands.

The ttSSB wild-type (WT) band gradually disappeared and shifted to the well edge in a ttRecO-concentration-dependent manner, indicating positively charged ttRecO captures ttSSB (Fig. 1B). The band was completely shifted at a ttSSB:ttRecO molar ratio of 1:2. As a control, substituting lysozyme for ttRecO caused no mobility shift in the ttSSB band even at a 1:3 molar ratio, indicating that ttSSB interacts specifically with ttRecO as previously reported *(**14**,*  *19**)*. The ttSSBΔIDL derivative exhibited a migration profile similar to that of ttSSB upon titration with ttRecO. In contrast, the bands of ttSSBΔC17 and ttSSBΔIDLΔC17—both lacking the C-tip region known to bind ttRecO *(**14**)*—shifted in a ttRecO-concentration-dependent manner but did not disappear, unlike ttSSB and ttSSBΔIDL. Notably, these shifts occurred at lower concentrations of ttRecO than those required for ttSSB and ttSSBΔIDL. In addition, ttSSBΔC17 and ttSSBΔIDLΔC17 showed slight shifts upon addition of lysozyme, implying reduced specificity for ttRecO. Taken together, these results suggest that ttSSB interacts with ttRecO not only via the C-tip but also via the OB-fold; however, the migration patterns of the shifted bands were markedly different depending on the presence or absence of the C-tip.

To clarify the contributions of each domain, we next constructed ProS2-fused ttSSB derivatives (ProS2-ttSSB, ProS2-OB_ttSSB_, ProS2-IDL_ttSSB_ and ProS2-C17_ttSSB_) and assessed their interactions with ttRecO (Fig. S2 and Fig. 1C). The ProS2 tag protein (pI = 5.43 and molecular mass = 23 kDa) and ProS2-IDL_ttSSB_ (pI = 5.88) showed no mobility shift even at high concentrations of ttRecO (Fig. S2A and Fig. 1C). Conversely, the bands of ProS2-ttSSB (pI = 5.40) and ProS2-C17_ttSSB_ (pI = 4.86) shifted and disappeared in a ttRecO-concentration-dependent manner (Fig. 1B and C). The ProS2-OB_ttSSB_ band (pI = 6.00) shifted upon titration with ttRecO, as observed for ttSSBΔC17 and ttSSBΔIDLΔC17. Collectively, these results indicate that both the OB-fold and the C-tip of ttSSB contribute to the interaction with ttRecO.

### Arg127 of ttRecO is essential for binding to the C-tip but not to the OB-fold of ttSSB

To further clarify the interaction sites between ttSSB and ttRecO, we constructed a site-directed mutant, ttRecO^R127A^, in which Arg127 was substituted with alanine. It has been reported that the C-tip of ttSSB binds to the Arg127 residue of ttRecO *(**14**)*.

**Fig. 2:**
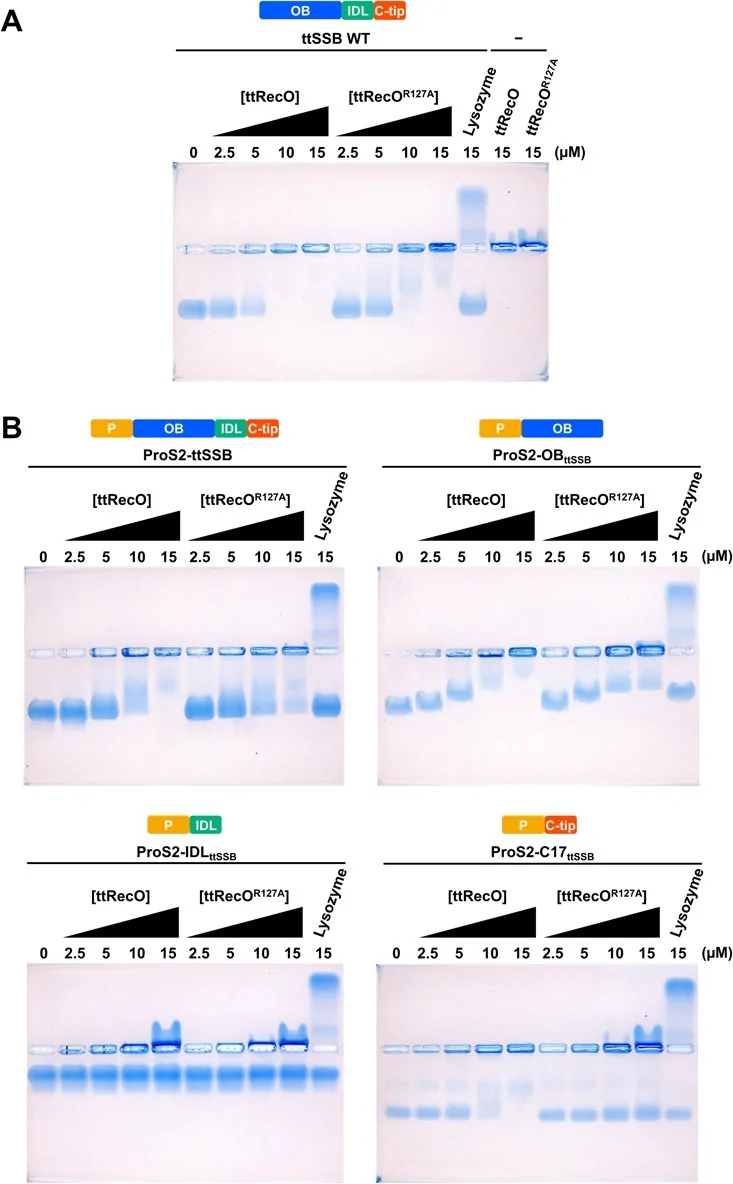
**Interactions between ttSSB derivatives and ttRecO**  ^**R127A**^  **assessed by native agarose gel electrophoresis**. ttSSB WT was analysed in (A) and ProS2-fused proteins (ProS2-ttSSB, ProS2-OB_ttSSB_, ProS2-IDL_ttSSB_, ProS2-C17_ttSSB_) were analysed in (B). The protein concentration of ttSSB and its derivatives was 5.0 μM, and ttRecO or ttRecO^R127A^ was added at the indicated concentrations. Lysozyme (15 μM) was used as a negative control.

Interactions between ttSSB derivatives and ttRecO^R127A^ were evaluated by native agarose gel electrophoresis (Fig. 2). As controls, we confirmed that the ProS2 tag protein and ProS2-IDL_ttSSB_ showed no interaction with ttRecO^R127A^ (Fig. S2B and Fig. 2B). In contrast to ttRecO WT, the ttSSB band did not completely disappear even at a ttSSB:ttRecO^R127A^ molar ratio of 1:3, indicating that the binding affinity was slightly reduced by the R127A mutation in ttRecO (Fig. 2A). Similar profiles were observed for ProS2-ttSSB and ProS2-OB_ttSSB_ (Fig. 2B). By contrast, the mobility shift of ProS2-C17_ttSSB_ was nearly absent in the presence of ttRecO^R127A^, which was entirely distinct from the profile observed with ttRecO. These results indicate that Arg127 of ttRecO constitutes a primary binding site for the C-tip of ttSSB and may also engage in interaction with the OB-fold of ttSSB.

### Effects of ttSSB on the dsDNA-binding activity of ttRecO

**Fig. 3:**
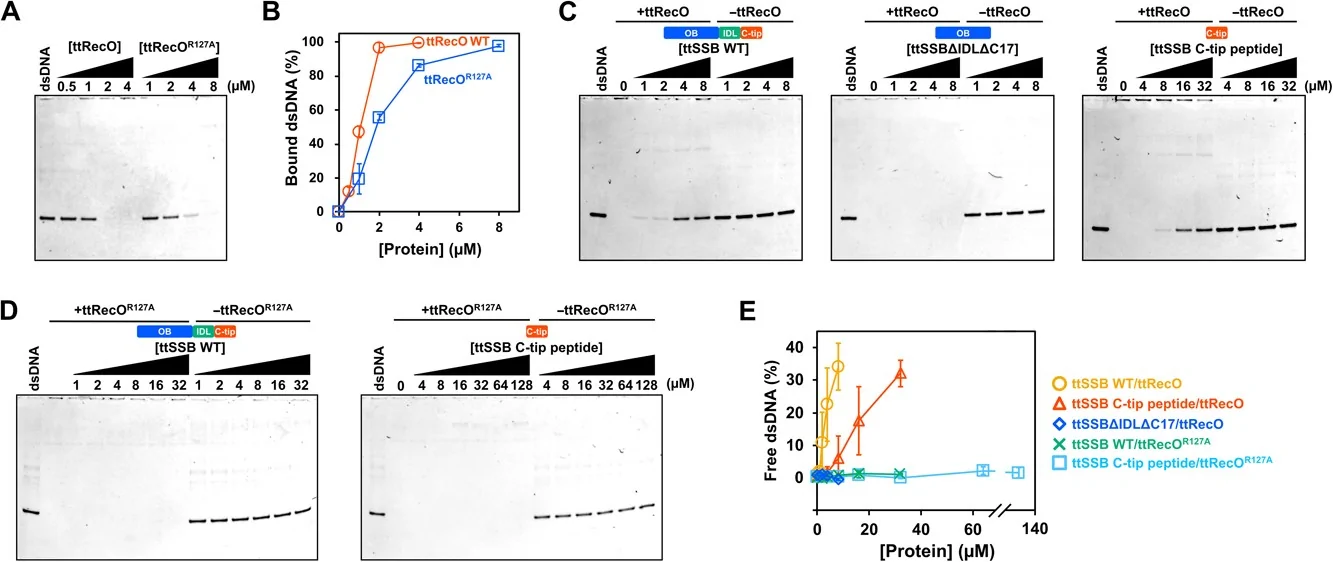
**Effects of ttSSB on the dsDNA-binding activity of ttRecO**. (A, B) Electrophoretic mobility shift assay of ttRecO and ttRecO^R127A^ binding to dsDNA. Reaction mixtures contained dsDNA (2 μM in bp) and the indicated concentrations of the proteins. A representative gel is shown in (A). Quantitative results for the protein-concentration dependence in DNA binding from three independent experiments are shown in (B). The percentage of bound dsDNA (%) was calculated as: Bound dsDNA (%) = 100 × (1 − *S*_free_/*S*_control_), where *S*_control_ is the dsDNA signal intensity in protein-free control and *S*_free_ is the free dsDNA signal intensity in each lane. Data are presented as mean ± SD (*n* = 3). (C–E) Effects of ttSSB on the dsDNA binding activities of ttRecO or ttRecO^R127A^ assessed by electrophoretic mobility shift assay. Reaction mixtures contained dsDNA (2 μM in bp), either 2.5 μM ttRecO or 8.5 μM ttRecO^R127A^, and the indicated concentrations of ttSSB and its derivatives. Reaction mixtures were prepared by first adding the buffer, followed by independent addition of protein/peptide and DNA solutions without premixing. Reactions were initiated by brief centrifugation to ensure simultaneous mixing of all components. Representative gels for ttRecO and ttRecO^R127A^ are shown in (C) and (D), respectively. Quantitative results for the concentration dependence of ttSSB and its derivatives in DNA binding from three independent experiments are shown in (E). The percentage of free dsDNA was calculated as: Free dsDNA (%) = 100 × *S*_free_/*S*_control_.

To gain functional insight into the ttSSB–ttRecO interaction sites, we examined how ttSSB affects the dsDNA-binding activity of ttRecO (Fig. 3). The 100-bp dsDNA band disappeared upon titration of ttRecO, indicating the reduced or altered electrophoretic mobility of dsDNA upon complex formation with the positively charged ttRecO (Fig. 3A and B). Even in the presence of 2.5 μM ttRecO, a free dsDNA band reappeared upon titration with ttSSB, suggesting that ttSSB suppresses the dsDNA-binding activity of ttRecO (Fig. 3C and E). This suppressive effect was not observed upon addition of ttSSBΔIDLΔC17 but was observed upon addition of the synthetic ttSSB C-tip peptide. Moreover, ttSSB exerted a stronger suppressive effect than the C-tip peptide. These data indicate that the ttSSB C-tip, rather than the OB-fold, suppresses the dsDNA-binding activity of ttRecO.

Comparison of the DNA binding profiles of ttRecO and ttRecO^R127A^ showed that the mutant displayed a ~2-fold reduction in dsDNA-binding activity relative to WT (Fig. 3A and B). In the presence of 8.5 μM ttRecO^R127A^, a condition that fully shifted dsDNA, addition of ttSSB or its C-tip peptide failed to suppress the dsDNA-binding activity even at high concentrations of 32 and 128 μM, respectively (Fig. 3D and E). These results suggest that Arg127 mediates both ttRecO dsDNA binding and its suppression by the ttSSB C-tip.

### *ttSSB domains responsible for formation of the ttSSB*–*ttRecO*–*ttRecR complex*

**Fig. 4:**
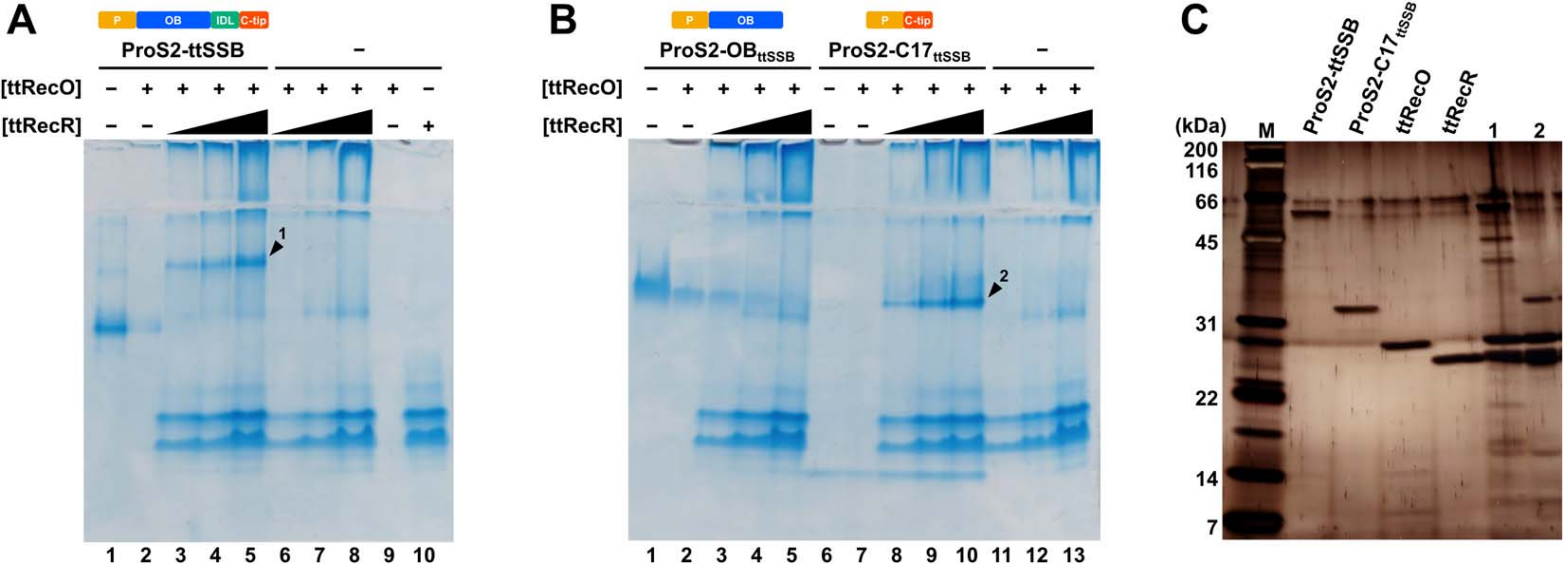
**Analysis of the ttSSB domains responsible for ttSSB–ttRecO–ttRecR complex formation**. (A and B) Complex formation was assessed by native PAGE. Reaction mixtures contained ProS2-fused proteins (ProS2-ttSSB, ProS2-OB_ttSSB_ and ProS2-C17_ttSSB_) at 5.0 μM, ttRecO at 15 μM and ttRecR at 7.5–30 μM. Arrows 1 and 2 indicate discrete bands excised for subsequent SDS-PAGE analysis. (C) SDS-PAGE analysis of the two gel pieces containing the bands indicated by the arrows 1 and 2 in (A and B). Protein bands were visualized by silver staining.

We assessed the contributions of individual ttSSB domains to assembly of the ttSSB–ttRecO–ttRecR complex by native PAGE (Fig. 4, Fig. S3A and B). The negatively charged ProS2-ttSSB, ProS2-OB_ttSSB_, ProS2-C17_ttSSB_ and ttRecR (pI = 5.43) migrated into the gel (Fig. 4A, lanes 1 and 10; Fig. 4B, lanes 1 and 6), whereas the positively charged ttRecO did not enter the gel (Fig. 4A, lane 9). Formation of the ternary complex was evaluated by comparing bands arising upon mixing all three components with those observed in the corresponding pairwise mixtures.

When ProS2-ttSSB and ttRecO were mixed at a 1:3 molar ratio and ttRecR was subsequently added at 0.5–2 molar equivalents relative to ttRecO, a discrete band appeared in the three-component mixtures (Fig. 4A, lanes 3–5). This band was absent from the ProS2-ttSSB/ttRecO mixture lacking ttRecR (Fig. 4A, lane 2) and from the ttRecO/ttRecR mixtures lacking ProS2-ttSSB (Fig. 4A, lanes 6–8). Likewise, with ProS2-C17_ttSSB_, a discrete band emerged in the three-component mixture (Fig. 4B, lanes 8–9) that was absent from both the ProS2-C17_ttSSB_/ttRecO mixture (Fig. 4B, lane 7) and the ttRecO/ttRecR mixtures (Fig. 4B, lanes 11–13). By contrast, no such band was detected in the ProS2-OB_ttSSB_/ttRecO/ttRecR mixtures (Fig. 4B, lanes 1–5). Nevertheless, the band derived from ProS2-OB_ttSSB_ appeared to slightly diminish in a ttRecR concentration–dependent manner in the presence of a fixed amount of ttRecO, implying a weak interaction between ProS2-OB_ttSSB_ and the ttRecO–ttRecR complex.

To verify the composition of the distinct bands, the corresponding gel regions were excised and crushed, followed by analysis by SDS-PAGE and silver staining (Fig. 4C). The distinct band from the ProS2-ttSSB/ttRecO/ttRecR mixture (Fig. 4A, lane 5, arrow 1) resolved into separate bands corresponding to ProS2-ttSSB, ttRecO and ttRecR (Fig. 4C, lane 1). Similarly, the distinct band from the ProS2-C17_ttSSB_/ttRecO/ttRecR mixture (Fig. 4B, lane 10, arrow 2) contained ProS2-C17_ttSSB_, ttRecO and ttRecR (Fig. 4C, lane 2). Taken together, these findings indicate that stable assembly of the ttSSB–ttRecO–ttRecR ternary complex is mediated by the C-tip of ttSSB, although a contribution from the OB-fold of ttSSB could not be substantiated by the present data.

### Effects of ttSSB on the ssDNA annealing activity of ttRecO

**Fig. 5:**
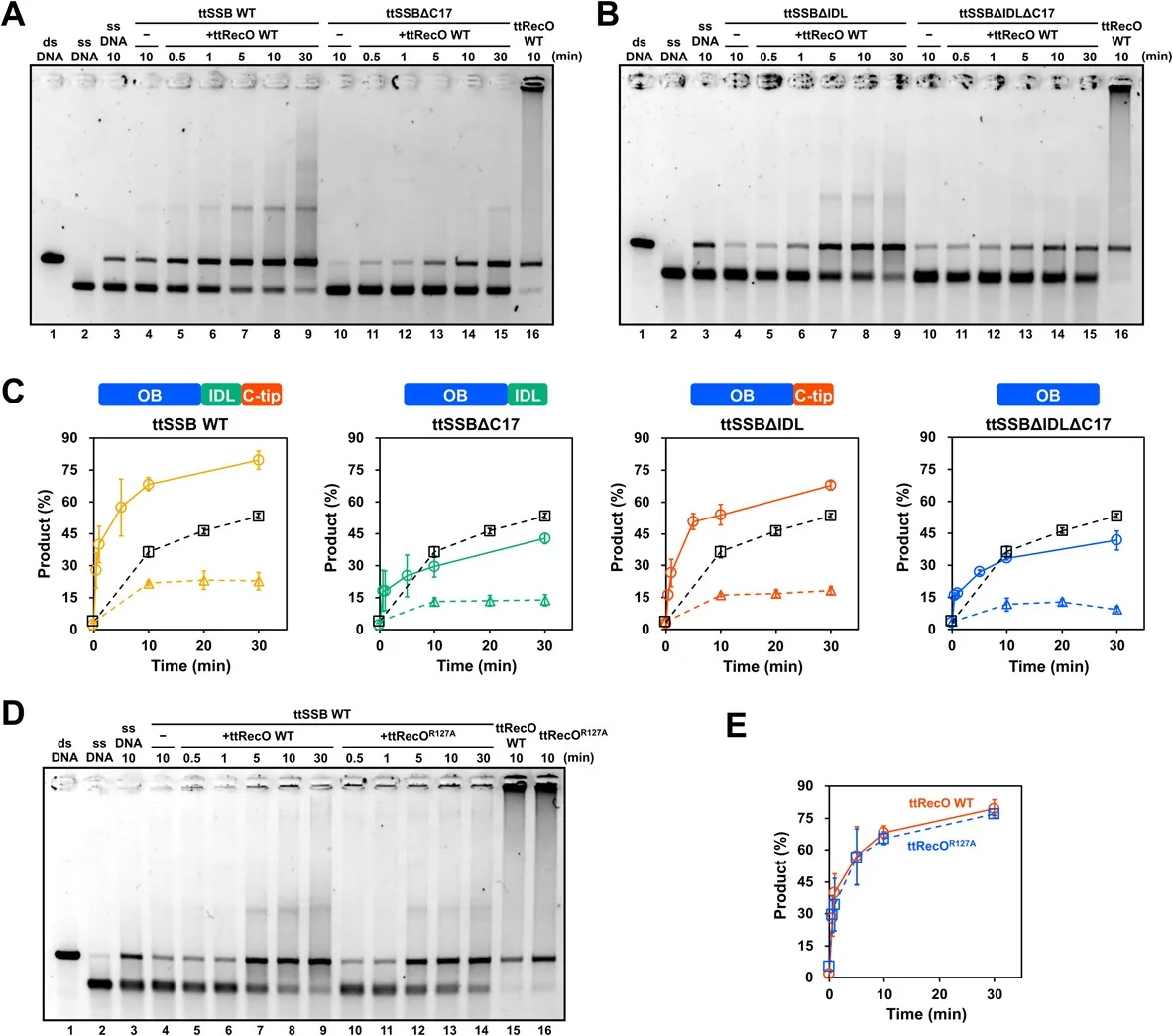
**Effects of ttSSB on the ssDNA annealing activity of ttRecO** . (A–C) ssDNA annealing assay of ttRecO in the presence of ttSSB and its derivatives. Reaction mixtures were prepared by first adding the buffer, followed by ssDNA and ttSSB solutions. After pre-incubation at 37°C for 5 min, reactions were initiated by the addition of ttRecO. After incubation at 37°C for the indicated times, reactions were stopped by the addition of SDS and proteinase K solutions, and then, the DNA products were analysed by agarose gel electrophoresis. Representative gels are shown in (A and B). Quantitative results from three independent experiments are shown in (C). The results for ttSSB WT (yellow), ttSSBΔC17 (green), ttSSBΔIDL (orange) and ttSSBΔIDLΔC17 (blue) are shown from left to right, respectively. Open circles with solid lines indicate reactions with ttRecO, open triangles with dotted lines indicate reactions without ttRecO, and open rectangles with black dotted lines indicate spontaneous ssDNA annealing. The percentage of annealed product was calculated as: Product (%) = 100 × *S*_dsDNA_/(*S*_ssDNA_ + *S*_dsDNA_), where *S*_ssDNA_ and *S*_dsDNA_ are the ssDNA and dsDNA signal intensities in each lane, respectively. Data are presented as mean ± SD (*n* = 3). (D, E) ssDNA annealing assay of ttRecO^R127A^ in the presence of ttSSB. A representative gel is shown in (D). Quantitative results from three independent experiments are shown in (E). Open circles with solid lines (orange) indicate reactions with ttRecO, whereas open rectangles with dotted lines (blue) indicate reactions with ttRecO^R127A^.

To elucidate the effects of ttSSB domains on the ssDNA annealing activity of ttRecO, we performed ssDNA annealing assays using heat-denatured linearized pUC119 dsDNA (Fig. 5). In the absence of protein, spontaneous ssDNA annealing was observed after 10 min at 37°C (Fig. 5A and B, lane 3). In the presence of ttRecO, nearly all ssDNA was converted into dsDNA or higher-order DNA aggregates, confirming that ttRecO possesses ssDNA annealing activity (Fig. 5A and B, lane 16). Conversely, in the presence of ttSSB and its derivatives, the spontaneous ssDNA annealing was prevented (Fig. 5A–C). Upon addition of ttRecO, ssDNA was converted to dsDNA even in the presence of ttSSB in a time-dependent manner, with ~80% of ssDNA converted to dsDNA within 30 min.

Meanwhile, in the presence of ttSSBΔC17 or ttSSBΔIDLΔC17, the ssDNA annealing activity of ttRecO was markedly reduced, and the conversion from ssDNA to dsDNA at 30-min incubation was limited to ~43 and ~42%, respectively (Fig. 5C). For ttSSBΔIDL, the decrease in annealing activity was modest, with ~68% of ssDNA converted to dsDNA at 30-min incubation. These data suggest that the interaction between the ttSSB C-tip and ttRecO alleviates the inhibitory effect of ttSSB on the ssDNA annealing activity of ttRecO. However, there remains the possibility that deletion of the C-tip alters the ssDNA-binding mode of ttSSB, thereby inhibiting annealing. Indeed, after 30 min, spontaneous ssDNA annealing was 23% with ttSSB and 18% with ttSSBΔIDL, whereas it decreased to 14% with ttSSBΔC17 and 10% with ttSSBΔIDLΔC17 (Fig. 5C and Fig. S4). Electrophoretic mobility shift assays using a 60-mer oligo(dT) showed no clear differences in ssDNA-binding affinity among the ttSSB derivatives (Fig. S5). However, differences in supershift patterns were observed at higher protein concentrations, depending on the presence or absence of the C-tip.

To clarify whether the ttSSB C-tip directly affects the ttRecO activity, we next conducted ssDNA annealing assays using ttRecO^R127A^, which lacks interaction with the ttSSB C-tip. ttRecO^R127A^ also catalysed ssDNA annealing in the absence of ttSSB, with nearly all ssDNA converted into dsDNA or aggregates (Fig. 5D, lane 16). In the presence of ttSSB, ssDNA was converted to dsDNA upon addition of ttRecO^R127A^, with ~77% of ssDNA converted within 30 min (Fig. 5D and E). These results indicate that the annealing efficiency on ttSSB-coated ssDNA is largely comparable between ttRecO and ttRecO^R127A^. Thus, the alleviation of the inhibitory effect of ttSSB on ttRecO-dependent ssDNA annealing in the presence of the C-tip is likely caused by C-tip-dependent changes in the ssDNA-binding mode of ttSSB, rather than by a direct stimulatory interaction with ttRecO.

This observation appears to be inconsistent with a previous study *(**14**)*, which reported that ttRecO^R127A^ exhibits no ssDNA annealing activity in the presence of ttSSB. Notably, the reaction buffers differed: our buffer contained no potassium or magnesium ions. We therefore repeated the assay under conditions closely matching the previous study. Under those ionic conditions, ttRecO^R127A^ showed no ssDNA annealing activity in the presence or even absence of ttSSB (Fig. S6), suggesting that, in the presence of potassium and magnesium ions, ttRecO^R127A^ loses its intrinsic annealing activity.

## Discussion

The interaction between SSB and RecO is central to understanding the molecular mechanisms of recombination mediators. To date, most studies have focused on contacts mediated by the conserved SSB C-tip *(**7**,*  *14**,*  *20**)*. In addition to confirming the established interaction between the C-tip of ttSSB and ttRecO, our work demonstrates that the OB-fold of ttSSB can directly bind ttRecO (Fig. 1). We cannot fully exclude the possibility that the observed electrophoretic mobility shift reflects non-specific contacts or electrostatic effects, rather than stable complex formation in native gels. However, a previous NMR study reported distinct chemical-shift perturbation patterns for the full-length ttSSB and the synthetic C-tip peptide, indirectly supporting a role for the OB-fold in contacting ttRecO *(**14**)*. Similarly, in *E. coli*, differences in the thermodynamic parameters from isothermal titration calorimetry measurements for RecO binding to the full-length SSB versus the C-tip peptide suggest a direct interaction between the SSB OB-fold and RecO *(**21**)*.

Although we were not able to establish the functional consequences of this ttSSB OB-fold–ttRecO interaction in the present study, a direct contact between the ssDNA-binding OB-fold of ttSSB and ttRecO is conceptually compatible with a handoff mechanism in which ttRecO replaces or displaces ttSSB on ssDNA. This scenario is reminiscent of a recent structural study on the eukaryotic RPA–Rad52 complex, where Rad52 interacts directly with the ssDNA-binding site of RPA, thereby providing a plausible molecular pathway for ssDNA handoff *(**22**)*. However, the ssDNA annealing activity of ttRecO was suppressed by ttSSB (Fig. 5), in contrast to the stimulatory effect of RPA on Rad52 activity *(**22**)*. Thus, while a handoff mechanism remains a plausible model, the available data do not yet establish its functional relevance in the ttSSB–ttRecO system.

The differential effects of ttSSB and its variants on ttRecO-mediated ssDNA annealing are best explained by C-tip-dependent changes in ttSSB’s ssDNA-binding mode, rather than by a specific functional interaction between the C-tip and ttRecO (Fig. 5 and Fig. S5). In *E. coli* SSB, removal of the C-tip increases intrinsic ssDNA affinity and alters intersubunit cooperativity, consistent with C-tip-mediated autoinhibition of the OB-fold ssDNA-binding surface *(**23**)*. Consistently, ttRecO^R127A^ retained WT levels of ssDNA annealing activity (Fig. 5D and E). Nevertheless, this conclusion is condition-dependent and should be treated with caution. Indeed, our data showed that buffer composition markedly influences ttRecO ssDNA annealing activity; under certain ionic conditions, ttRecO^R127A^ loses activity (Fig. S6). It is noteworthy that this phenomenon may relate to its reduced dsDNA binding.

We further showed that the ttSSB C-tip engages Arg127 of ttRecO and, importantly, suppresses the dsDNA-binding activity of ttRecO (Fig. 3). While the C-tip binding to Arg127 is consistent with the previous report *(**14**)*, to our knowledge, the observation that ttSSB modulates dsDNA-binding activity of ttRecO has not been described previously, either in *T. thermophilus* or other organisms. Notably, ttRecO^R127A^ exhibited a ~2-fold reduction in dsDNA-binding activity relative to WT, and this C-tip-dependent suppression of dsDNA binding was completely attenuated in this mutant. Our data therefore implicate Arg127 of ttRecO in interaction with dsDNA and suggest that the binding of the ttSSB C-tip at or near this site competitively suppresses dsDNA binding by ttRecO. Given that SSB recruits RecO preferentially to ssDNA regions, it is reasonable that SSB would also steer RecO away from dsDNA, thereby sharpening substrate selectivity.

Previous studies on assembly of the SSB–RecO–RecR ternary complex have been limited to work in *E. coli* using the SSB C-tip peptide *(**20**)* and to functional assays in which RecOR acts on SSB-coated ssDNA *(**19**,*  *24**,*  *25**)*. To our knowledge, this study provides the first experimental evidence for the formation of the full-length SSB–RecO–RecR ternary complex and its detection by native PAGE (Fig. 4). The existence of such a stable ternary complex supports the previous functional observations of SSB–RecOR interplay on ssDNA and strengthens current mechanistic models of recombination mediators *(**19**,*  *24**,*  *25**)*.

SSB architecture and RecO amino acid sequences vary widely across bacteria: SSB is dimeric in *Thermus* and *Deinococcus* but tetrameric in *E. coli*; homology searches reveal low amino acid sequence identity (~20%) between *Thermus*/*Deinococcus* RecO and *E. coli* RecO; and the C-tip–RecO interaction sites differ across species *(**7**,*  *14**)*. Despite this diversity, the core functions attributed to recombination mediators appear broadly conserved. Continued cross-species analyses will be essential to delineate the conserved molecular mechanisms of recombination mediators. In particular, we anticipate that high-resolution structures of the SSB–RecO and SSB–RecO–RecR complexes will resolve outstanding questions regarding binding interfaces, allosteric regulation and ssDNA handoff mechanisms.

## Supplementary Material

Web_Material_mvag042
