# Supplementary material for: Involvement of the OB-fold and the C-terminal acidic tip of SSB in its interaction with RecO from Thermus thermophilus HB8
Source: J Biochem. 2026 Jun 16;180(3):231–41. doi: 10.1093/jb/mvag042 (PMC13489159; doi:10.1093/jb/mvag042)
Supplement: Supplementary file 1 [file web_material_mvag042.pdf]

Supplementary Data

Involvement of the OB-fold and the C-terminal acidic tip of SSB in its interaction with RecO from *Thermus thermophilus* HB8

Kazuki Kobayashi[1], Masao Inoue[1,2,*], Kenji Fukui[3], Riku Aono[1], Anna Ochi[1], Ryoji Masui[4], and Hisaaki Mihara[1,*]

[1]College of Life Sciences, Ritsumeikan University, 1-1-1 Nojihigashi, Kusatsu, Shiga 525-8577, Japan

[2]R-GIRO, Ritsumeikan University, 1-1-1 Nojihigashi, Kusatsu, Shiga 525-8577, Japan

[3]Department of Food Science and Nutrition, Nara Women's University, Kita-Uoya-Nishimachi, Nara, Nara 630-8506, Japan.

[4]Graduate School of Science, Osaka Metropolitan University, 3-3-138 Sugimoto, Sumiyoshi-ku, Osaka 558-8585, Japan

*Corresponding authors: Masao Inoue; mainoue@fc.ritsumei.ac.jp; R-GIRO, Ritsumeikan University, Kusatsu, Shiga 525-8577, Japan; Tel.: +81-77-599-4346; Hisaaki Mihara; mihara@fc.ritsumei.ac.jp; College of Life Sciences, Ritsumeikan University, Kusatsu, Shiga 525-8577, Japan; Tel.: +81-77-561-2732

Table S1. Sequences of primers used for this study

**Construct**	**Sequence**
pET-11a/tt*ssbΔC17*	5′-TAATAAGGATCCGGCTGCTAACAAAGCCCGAAAGG-3′
	5′-CACCCCACCCGTCTGGACTTCGCGGG-3′
pET-HisTEV/tt*ssb*<br>pET-HisTEV/tt*ssbΔC17*	5′-GTATTTTCAGGGACATATGGCTCGAGGCCTGAACCG-3′
	5′-CCTTTCGTCTTCAAGAATTCTCATGTTTGACAGC-3′
pET-HisTEV/tt*ssbΔIDL*	5′-CACCCGTGACATTGACGAAGGCTTGGAAGACTTTCC-3′
	5′-TCAATGTCACGGGTGGGGCGCT-3′
pET-HisTEV/tt*ssbΔIDLΔC17*	5′-CACCCGTTAATAAGGATCCGGCTGCTAACAAAGCC-3′
	5′-CCTTATTAACGGGTGGGGCGC-3′
pCold-ProS2TEV/tt*ssb*	5′-ATTTTCAGAGCCATATGGCTCGAGGCCTGAACCG-3′
	5′-AGATTACCTATCTAGATTATTAAAACGGCAACTCCTCCTCCGGC-3′
pCold-ProS2TEV/OB_{ttSSB}	5′-TAATAATCTAGATAGGTAATCTCTGCTTAAAAGC-3′
	5′-CTATCTAGATTATTAACGGGTGGGGCGC-3′
pCold-ProS2TEV/IDL_{ttSSB}	5′-TCTAGATAGGTAATCTCTGCTTAAAAGCAC-3′
	5′-ATGGCTCTGAAAATACAGGTTTTCC-3′
	5′-TATTTTCAGAGCCATGGACCTGCCCAGGC-3′
	5′-GATTACCTATCTAGATTATTACACCCCACCCGTC-3′
pCold-ProS2TEV/$C17_{ttSSB}$	5′-TATTTTCAGAGCCATGACATTGACGAAGGCTTGGAAG-3′
	5′-ATGGCTCTGAAAATACAGGTTTTCC-3′
pET-11a/tt*recO*^{R127A}	5′-GCCTCGCCGGGATCGCCAAGCACG-3′
	5′-CGATCCCGGCGAGGCCCGAGACCAGAAG-3′
100 bp dsDNA	5′-ATGAGTATTCAACATTTCCGTGTC-3′
	5′-CATCTTTTACTTTCACCAGCGT-3′

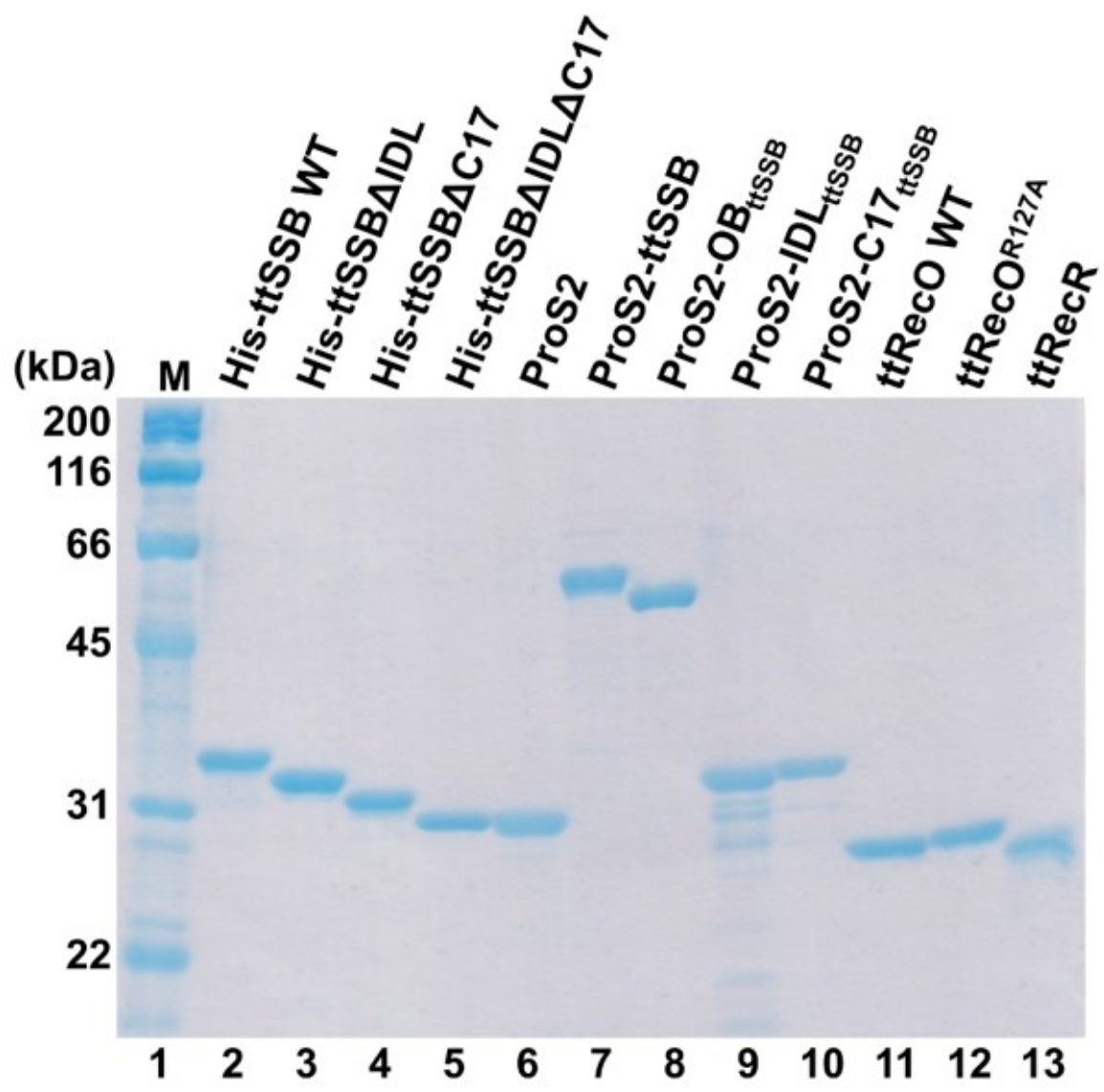

Fig. S1. Verification of purified proteins by SDS-PAGE. Each protein sample (1 μg) was separated on a 15% SDS-PAGE gel under reducing conditions and visualized by staining with Coomassie Brilliant Blue G-250. The lanes contained the following samples: 1, molecular mass markers (200, 116, 66, 45, 31 and 22 kDa); 2, ttSSB (32.3 kDa); 3, ttSSBΔIDL (30.6 kDa); 4, ttSSBΔC17 (30.3 kDa); 5, ttSSBΔIDLΔC17 (28.6 kDa); 6, ProS2 (21.2 kDa); 7, ProS2-ttSSB (54.6 kDa); 8, ProS2-OB_{ttSSB} (50.9 kDa); 9, ProS2-IDL_{ttSSB} (26.4 kDa); 10, ProS2-$C17_{ttSSB}$ (26.7 kDa); 11, ttRecO (24.8 kDa); 12, ttRecO^{R127A} (24.7 kDa); 13, ttRecR (21.2 kDa).

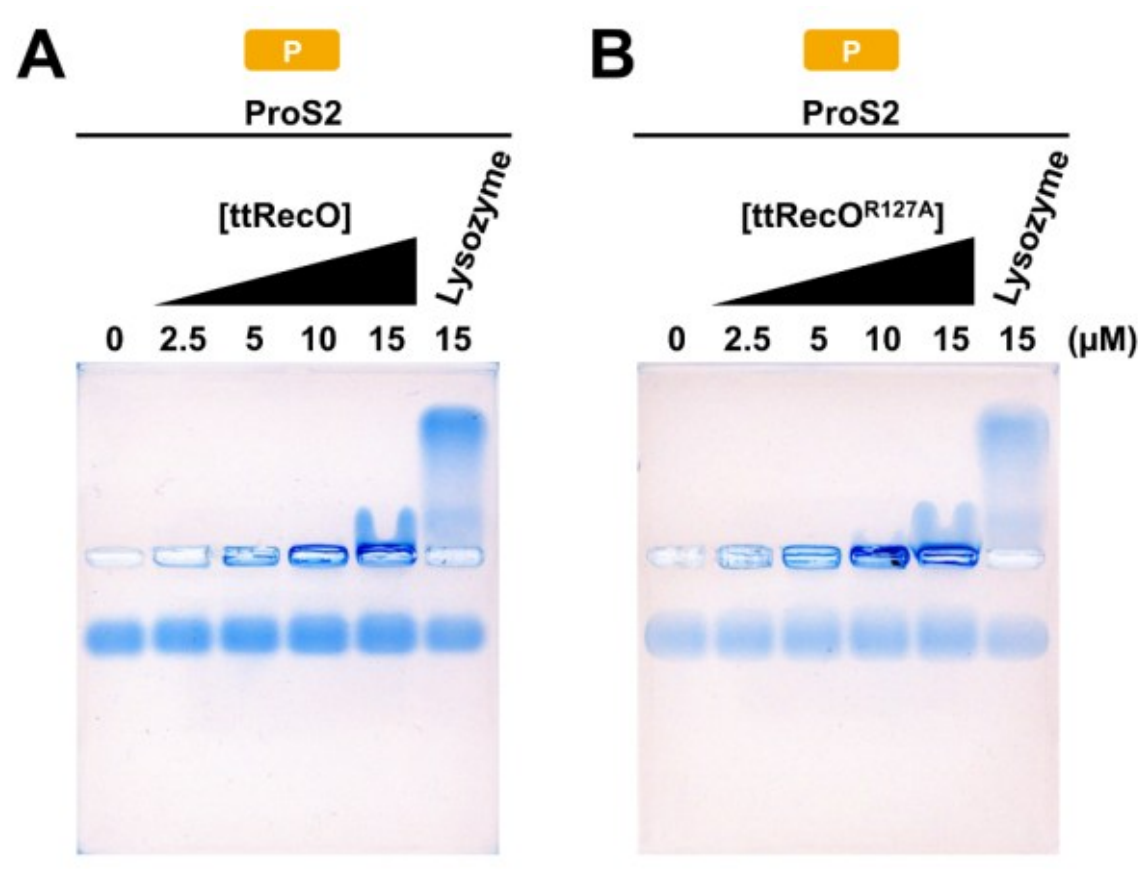

Fig. S2. Analysis of the interactions between ProS2 and ttRecO **(A)** or ttRecO^{R127A} **(B)**. The protein concentration of ProS2 was 5.0 μM, and ttRecO was added at the indicated concentrations.

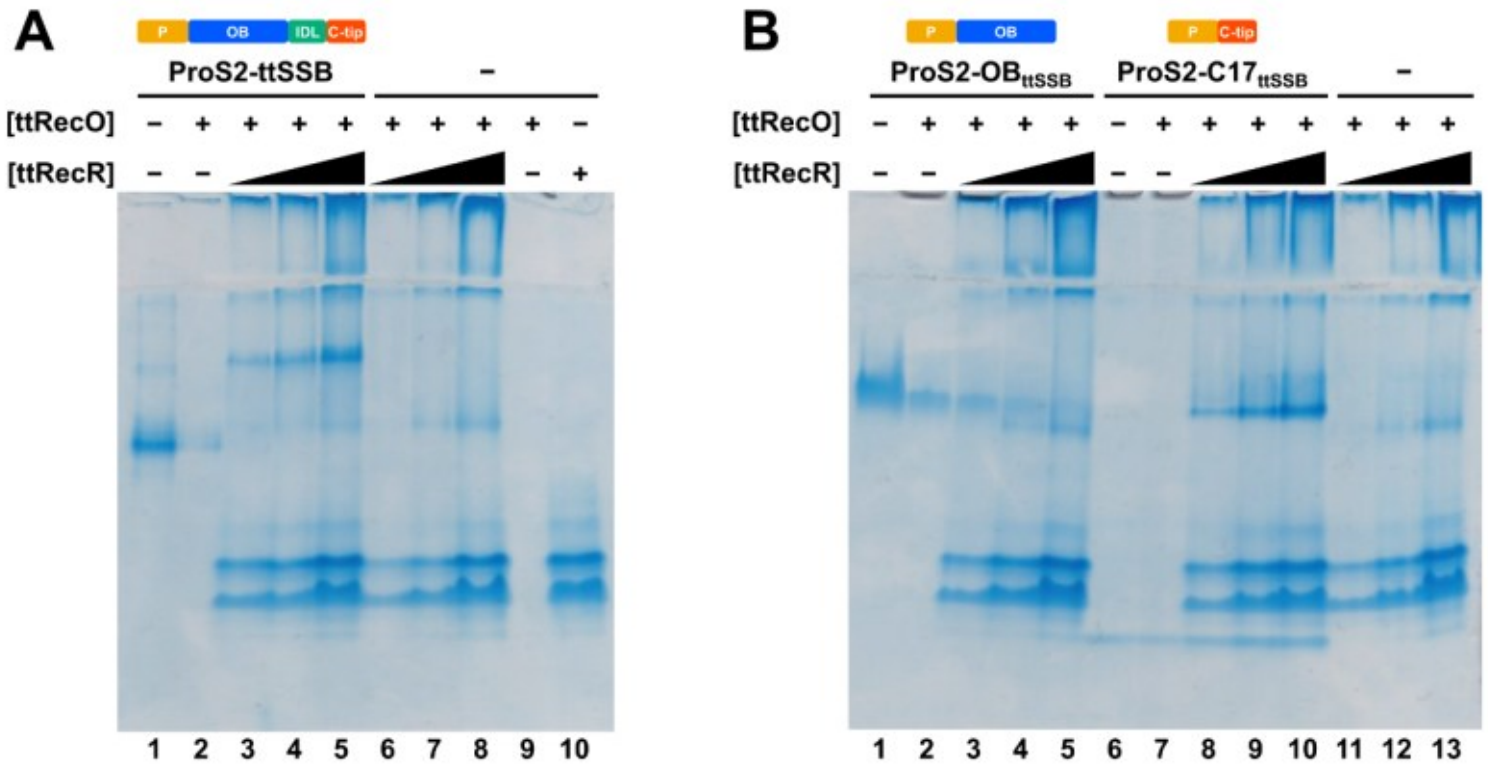

Fig. S3. Uncropped images of the gels (without arrows) shown in Fig. 5A and B.

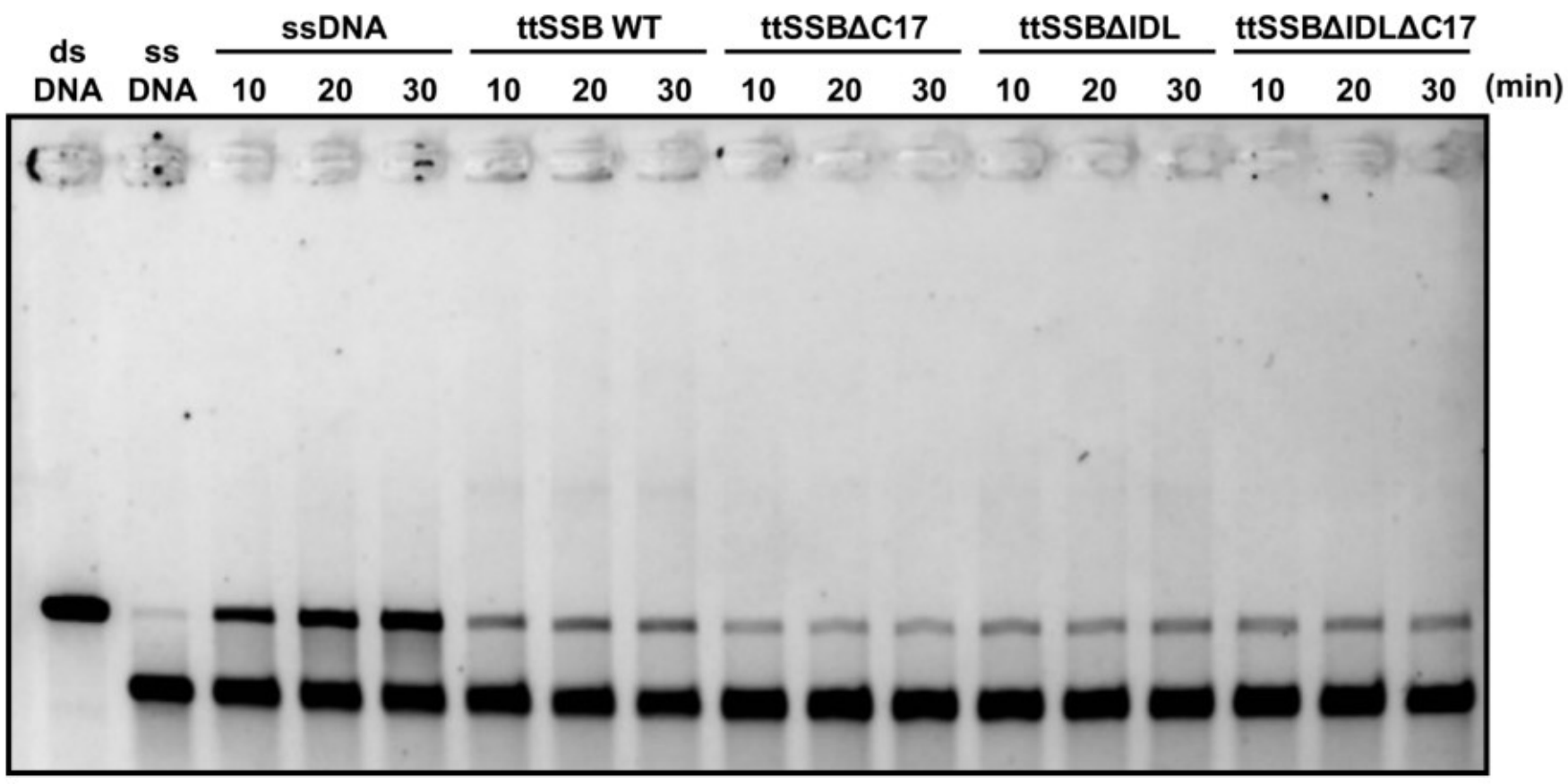

Fig. S4. ssDNA annealing in the absence of ttRecO. Either 15 μM(nt) ssDNA alone or mixed with a ttSSB domain-truncated mutant was incubated. Reactions were stopped at the indicated times, and the DNA products were analyzed by agarose gel electrophoresis.

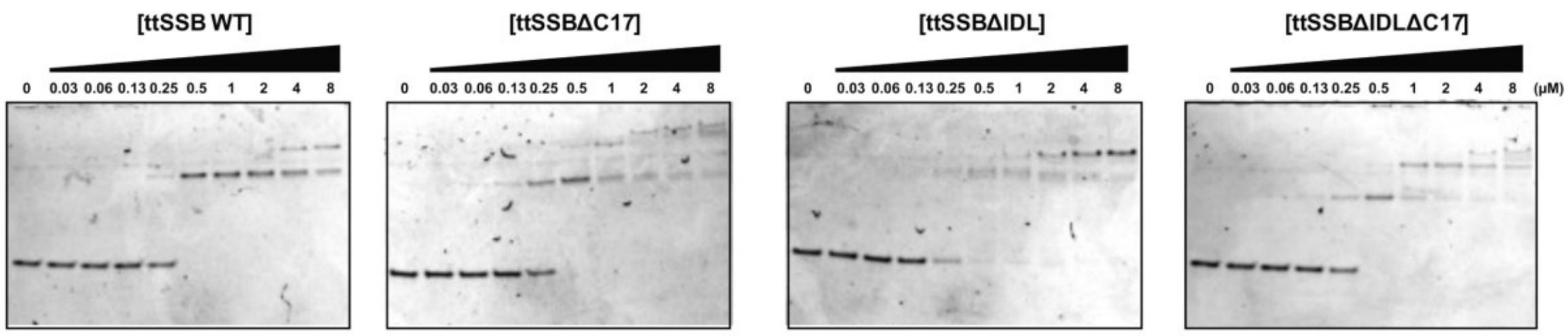

Fig. S5. Verification of ssDNA-binding activity of ttSSB and its derivatives by electrophoretic mobility shift assay using a 60-mer oligo(dT) (300 nM). ttSSB and its derivatives were added at the indicated concentrations.

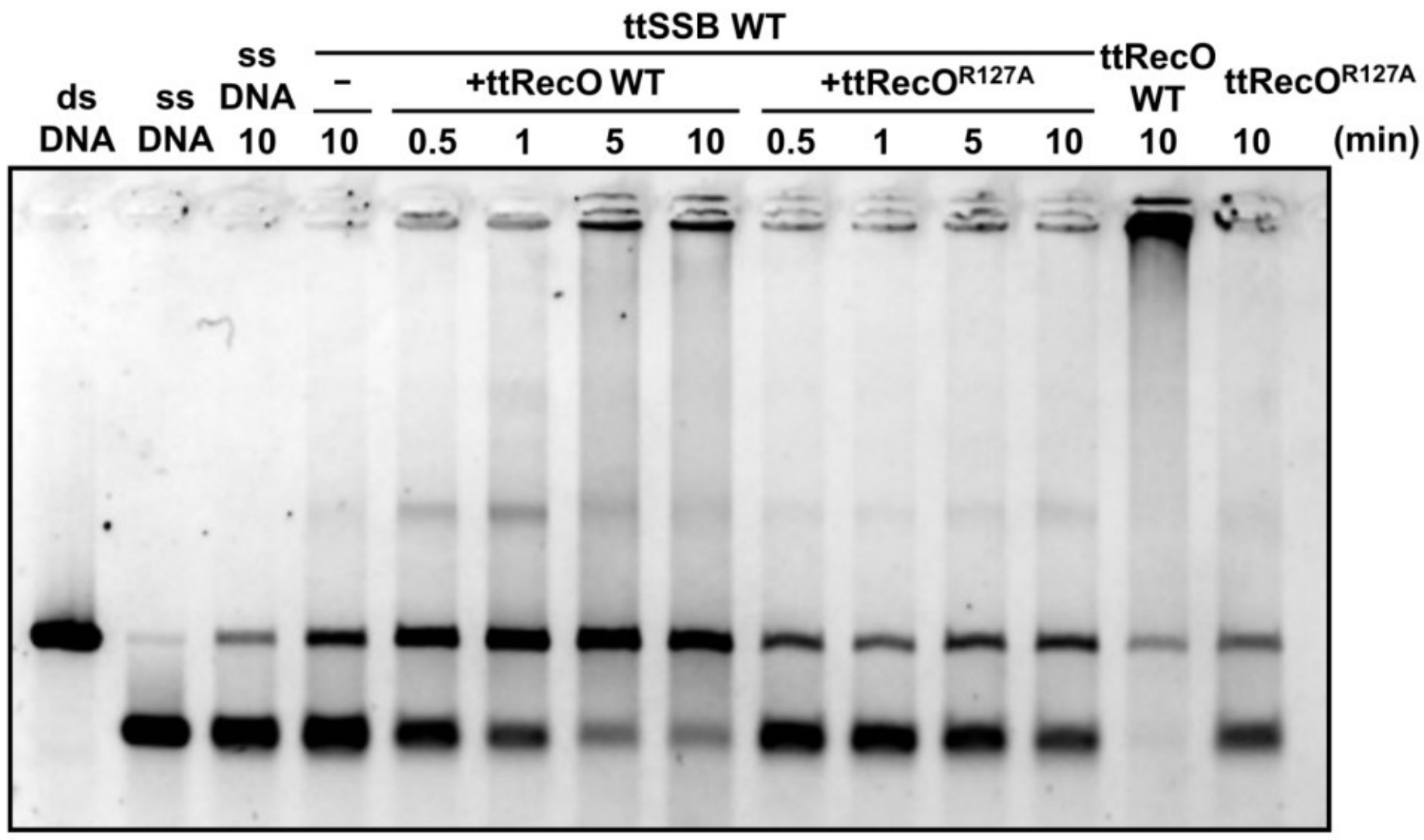

Fig. S6. Verification of ssDNA annealing activity of ttRecO and ttRecO^{R127A} in the buffer conditions of the previous study *(14)*.
